# Impact of metal implants on xSPECT/CT Bone reconstruction: the “shining metal artefact”

**DOI:** 10.1186/s41824-020-00087-7

**Published:** 2020-10-01

**Authors:** Thiago V. M. Lima, Ujwal Bhure, Maria de Sol Pérez Lago, Yannick Thali, Savo Matijasevic, Justus Roos, Klaus Strobel

**Affiliations:** 1 Radiologie und Nuklearmedizin, Luzerner Kantonsspital, Lucerne, Switzerland; 2 Institute of Radiation Physics, Lausanne University Hospital and University of Lausanne, Lausanne, Switzerland

**Keywords:** Bone SPECT/CT, xSPECT Bone, Flash3D, Metal implant, Reconstruction artefact, Shining metal artefact

## Abstract

**Background:**

Novel reconstruction algorithms, such as xSPECT Bone, are gaining more and more importance in Nuclear Medicine. With xSPECT Bone, the reconstructed emission image is enhanced by the information obtained in the corresponding CT image. The CT defines tissue classes according to the Hounsfield units. In the iterative reconstruction, each tissue class is handled separately in the forward projection step, and all together in the back projection step. As a consequence, xSPECT Bone reconstruction generates images with improved boundary delineation and better anatomic representation of tracer activity. Applying this technique, however, showed that artefacts may occur, when no uptake regions, like metal implants, exhibit fictitious uniform tracer uptake. Due to limitations in spatial resolution in gamma cameras, the xSPECT Bone reconstructed image resulted in spill-out activity from surrounding high uptake region being uniformly distributed over the metal implants.

This new technology of xSPECT Bone reconstruction in general enhances the image quality of SPECT/CT; however, the potential introduction of specific artefacts which inadvertently come along with this new technology and their frequency have not yet been addressed in the literature. Therefore, the purpose of this work was to identify and characterize these specific metal artefacts (the so-called shining metal artefact) in order to reduce false positives and avoid potentially misdiagnosing loosened or infected implants.

**Case presentation:**

In this work, we report five cases imaged with bone SPECT/CT of 5 anatomical regions (foot, elbow, spine, shoulder, ribs and knee). All cases demonstrated “shining metal artefacts” in xSPECT Bone reconstruction.

**Conclusion:**

While xSPECT Bone reconstruction algorithm significantly improves image quality for the diagnosis of bone and joint disorders with SPECT/CT, specific “shining metal artefacts” caused by the xSPECT Bone have to be recognized in order to avoid image misinterpretation suggesting metallic implant loosening or possible infection. The simultaneous analysis of conventionally reconstructed SPECT images (for Siemens the Flash3D reconstruction) helps to avoid misinterpretation of potential artefacts introduced by xSPECT Bone reconstruction.

## Background

Planar scintigraphy and single-photon emission computed tomography/computed tomography (SPECT/CT) are increasingly used and are indeed cost-effective advanced imaging techniques for the evaluation of bone and joint diseases (Gnanasegaran et al., 2009; Scharf, 2009; Van den Wyngaert et al., 2018a; Van den Wyngaert et al., 2016). SPECT/CT has been shown to be useful in the postoperative setting to evaluate healing or complications like infection, loosening or non-union after implantation of various metallic implants for arthrodesis, fracture stabilization or joint replacement with arthroplasties (Gnanasegaran et al., 2018; Kampen et al., 2018; Van den Wyngaert et al., 2018b; van der Bruggen et al., 2018).

SPECT iterative reconstruction is widely used in clinical practice and has contributed to significant improvement in image quality in the recent years. Much of the recent developments involved the extension of the system model to incorporate additional factors in order to more accurately model the emission/detection process (Hutton, 2011).

xSPECT Bone belongs to this group of new generation of SPECT reconstruction methods. With xSPECT Bone, the reconstructed emission volume is partitioned into segments defined by the Hounsfield units obtained from the CT image. Zones for air/lung tissue, adipose tissue, soft tissue, soft bone and cortical bone are defined. Each zone is handled separately in the forward projection step (volume ➔ projection) of the multimodal iterative reconstruction, whereas the backward projection step (projection ➔ volume) takes all the data together. The main implication is that by this approach, xSPECT Bone is capable of improving the final radiopharmaceutical uptake images with enhanced delineation of the boundaries and better anatomic representation of radiotracer activity (Duncan & Ingold, 2018; Vija, 2013).

In this work, we report five cases of different anatomic regions (foot, elbow, spine, shoulder, ribs and knee), where due to characteristics of the xSPECT Bone reconstruction there is fictitious radiotracer uptake in the metal hardware (arthroplasty, screws and plates) in the patients’ reconstructed images. This probably occurred because regions of increased radiotracer uptake partially overlapped the metal implants. Due to this overlap, the projected region statistics for the metal implants are likely to be greater than the threshold, as mentioned above, and therefore, the metal implant region is not ignored for the reconstruction. And since the counts are assumed to belong to the whole segmented region, metal implants become associated with significantly increased activity. Duncan et al. reported that the presence of these artefacts is rare (Duncan & Ingold, 2018). In the preliminary evaluation of our data, we observed the “shining metal artefact” in 11 of 47 cases (23.4%) of bone and joint SPECT/CT with metallic hardware. We encountered this artefact in various sizes of metal implants and all anatomic regions. Increased radiotracer activity around metallic implants is a sign for loosening or infection and is often accompanied by lucencies on the CT part of the study (Hudyana et al., 2016; Murer et al., 2020; Romer et al., 2005). The “shining metal artefact” might be misdiagnosed for loosening or infection if the reader is not aware of its presence and typical manifestation. Interestingly, in all of these cases, such artefact was not observed when the same images were processed using conventional Flash3D reconstruction algorithm and not accompanied by lucencies on CT.

## Case presentation

**Fig. 1:**
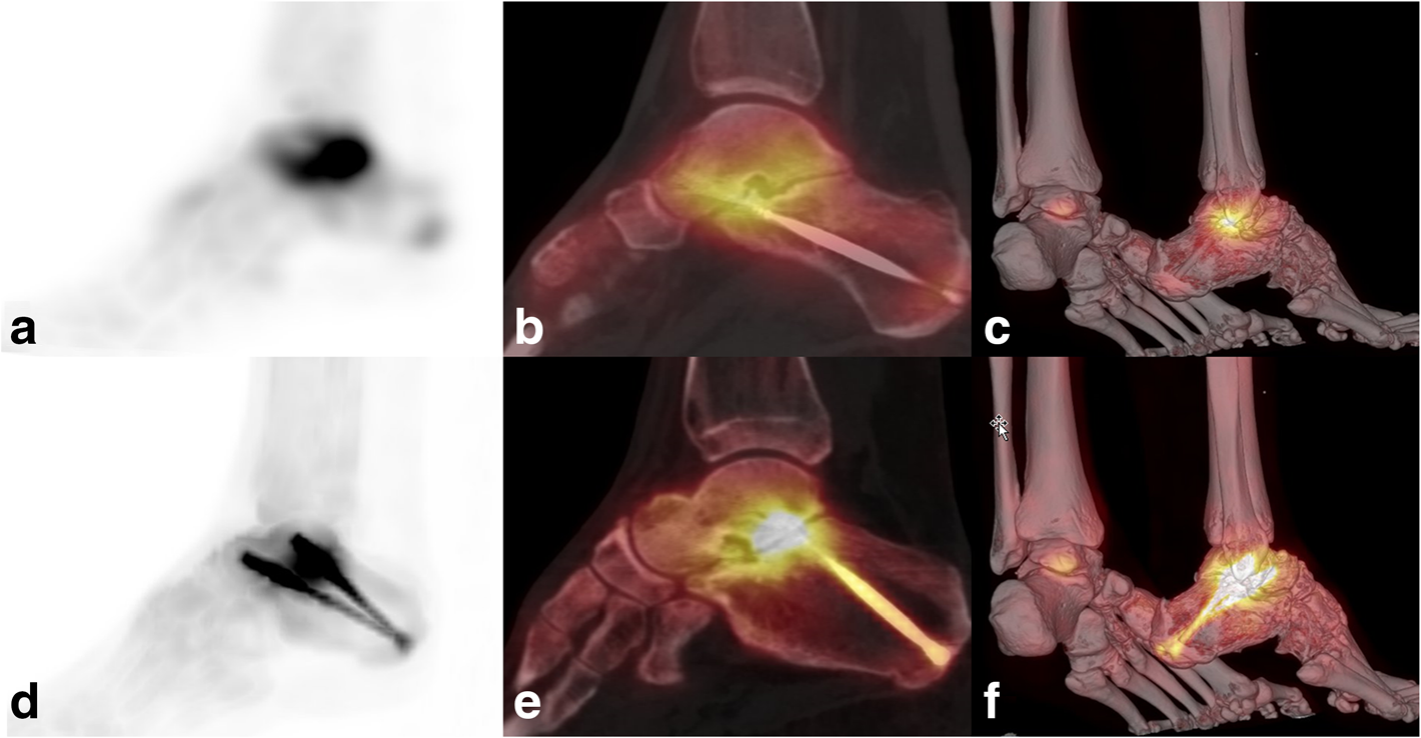
xSPECT Bone “shining metal artefact” in the foot. SPECT/CT images of a 35-year-old female patient with pain in the ankle 17 months after arthrodesis of the right lower ankle joint. MIP image with xSPECT Bone reconstruction (**d**), fused sagittal SPECT/CT (**e**) and fused 3D reconstruction (**f**) show high “pseudouptake” of the screws and true increased uptake in the non-union. Corresponding images (**a**–**c**) with conventional Flash3D reconstruction show no such uptake of the radiotracer around the screws and no morphologic signs of loosening with maintained true uptake of the non-union. Re-arthrodesis was performed as a consequence of the SPECT/CT results

**Fig. 2:**
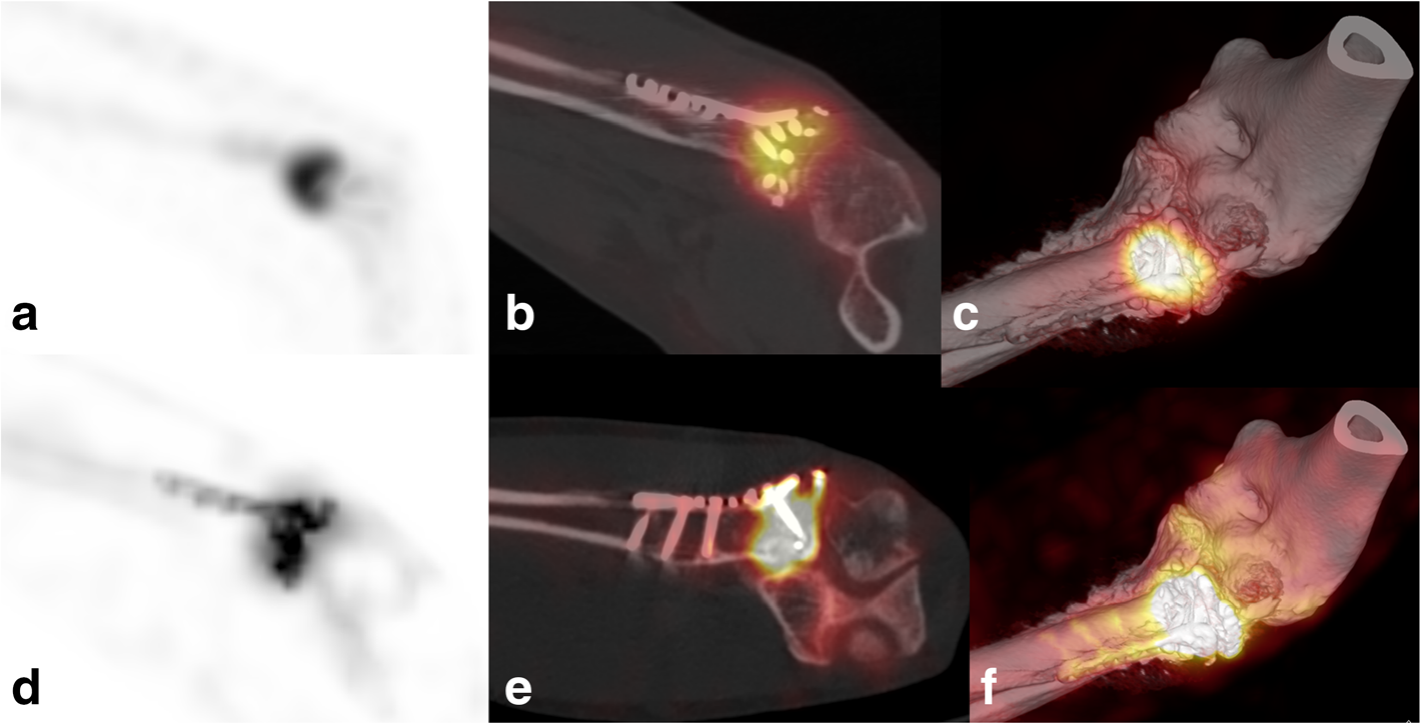
xSPECT Bone “shining metal artefact” in the elbow. SPECT/CT images of a 45-year-old male patient 6 months after osteosynthesis of a fracture of the proximal radius with persisting pain and suspicion for non-union. MIP image with xSPECT Bone reconstruction (**d**), fused sagittal SPECT/CT (**e**) and fused 3D reconstruction (**f**) show high “pseudouptake” of the plate and mini screws and true increased uptake in the non-union. Corresponding images (**a**–**c**) with conventional Flash3D reconstruction show no abnormal uptake of the radiotracer around the screws and no morphologic signs of loosening with maintained true uptake of the non-union. As a consequence of SPECT/CT, arthroplasty of the radial head was recommended

**Fig. 3:**
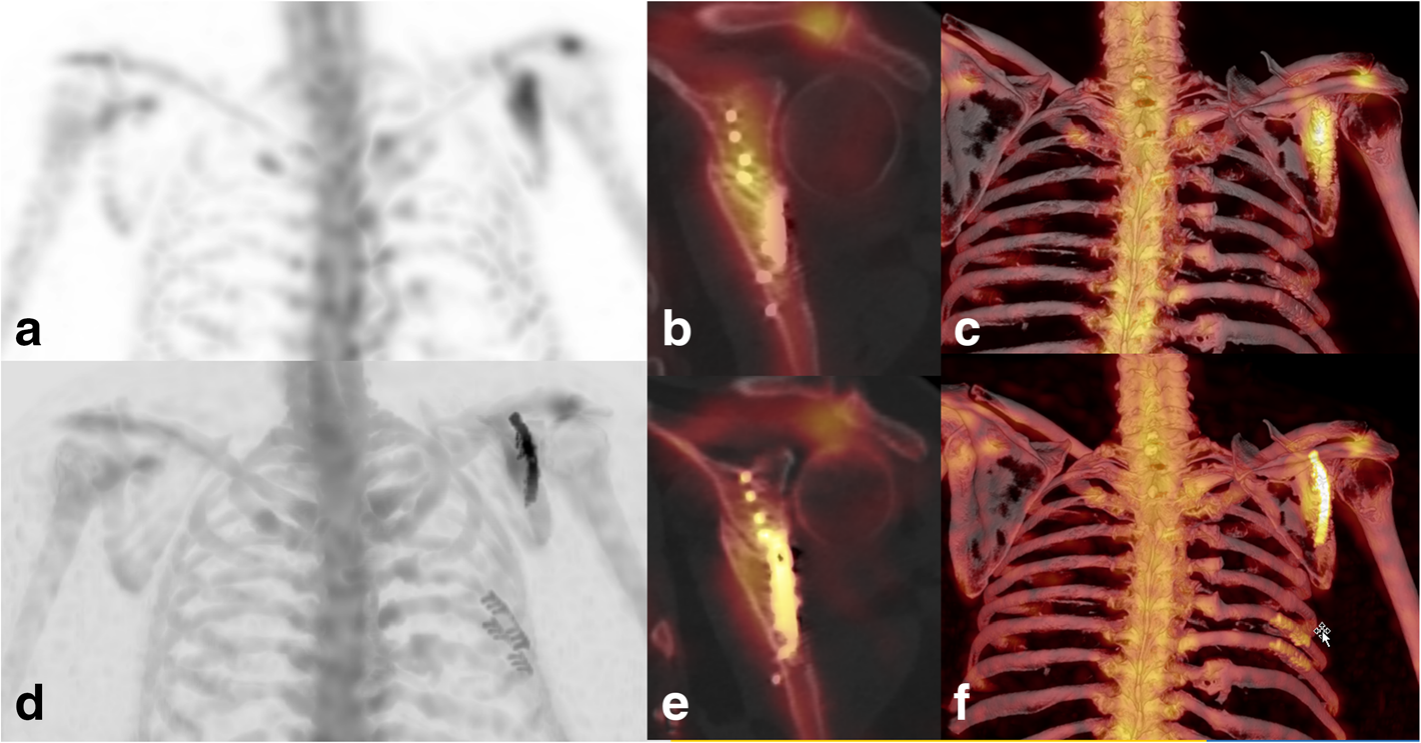
xSPECT Bone “shining metal artefact” in the shoulder. SPECT/CT images of a 57-year-old male patient with pain in the left shoulder 3 years after osteosynthesis of a scapula fracture and two ribs. MIP image with xSPECT Bone reconstruction (**d**), fused coronal SPECT/CT (**e**) and fused 3D reconstruction (**f**) show high “pseudouptake” of the plates and screws in the scapula and increased uptake around the non-union. “Pseudouptake” of the screws and plate in the ribs. Corresponding images (**a**–**c**) with conventional Flash3D reconstruction show no increased uptake of the radiotracer around the screws but active remodelling in the adjacent scapular bone around the non-union and no uptake in the ribs. Besides non-union, the patient suffered from neuropathic pain and conservative therapy was initiated

**Fig. 4:**
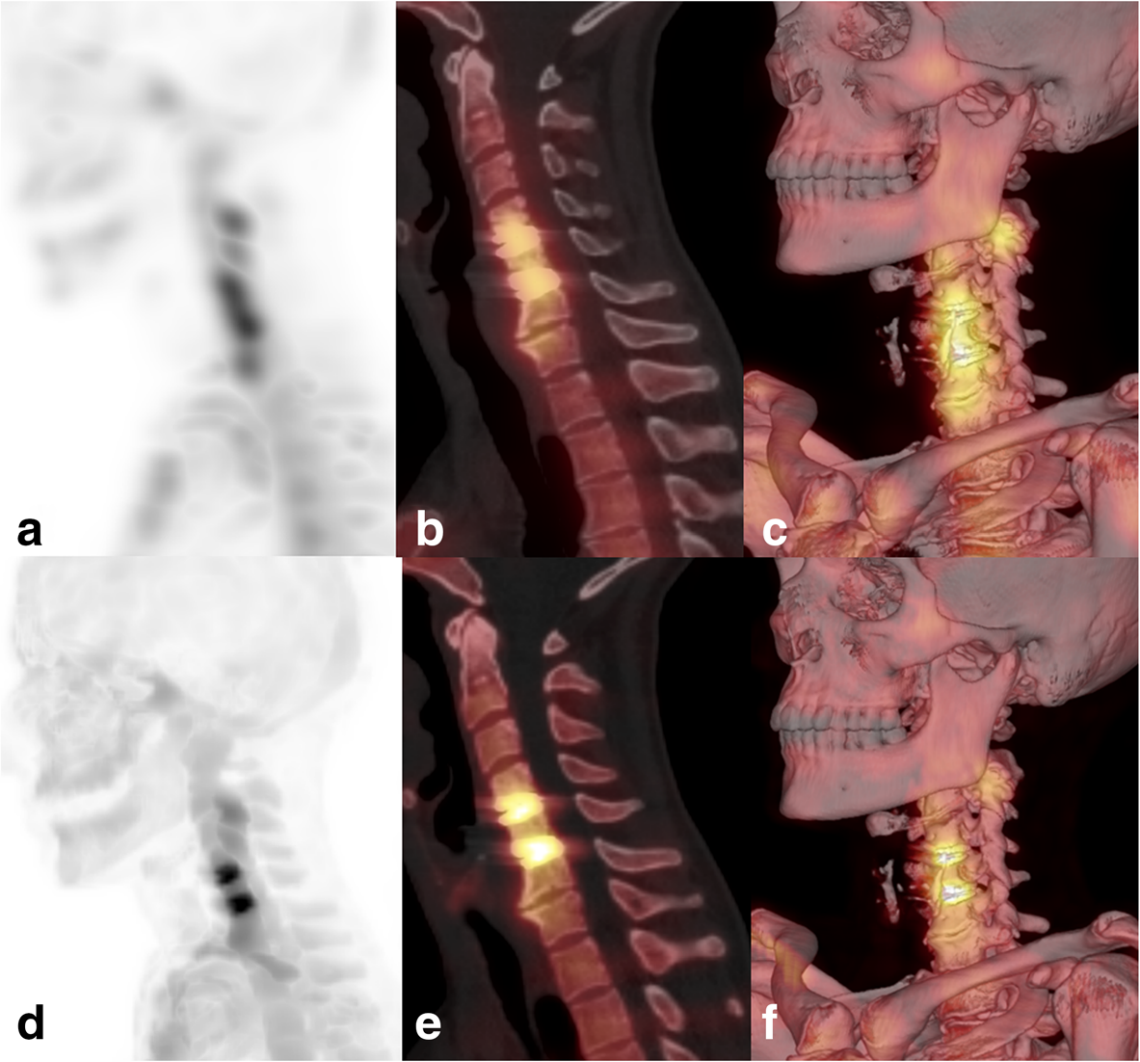
xSPECT Bone “shining metal artefact” in the cervical spine. SPECT/CT images of a 48-year-old female patient 10 months after operation of cervical spine with insertion of disc replacement devices. MIP image with xSPECT Bone reconstruction (**d**), fused sagittal SPECT/CT (**e**) and fused 3D reconstruction (**f**) show high “pseudouptake” of the disc replacements. Corresponding images (**a**–**c**) with conventional Flash3D reconstruction show no such increased uptake in the metal implants but increased uptake in the proximal vertebral bodies and facet joint osteoarthritis. Facet joint infiltration was performed as a consequence of the SPECT/CT results

**Fig. 5:**
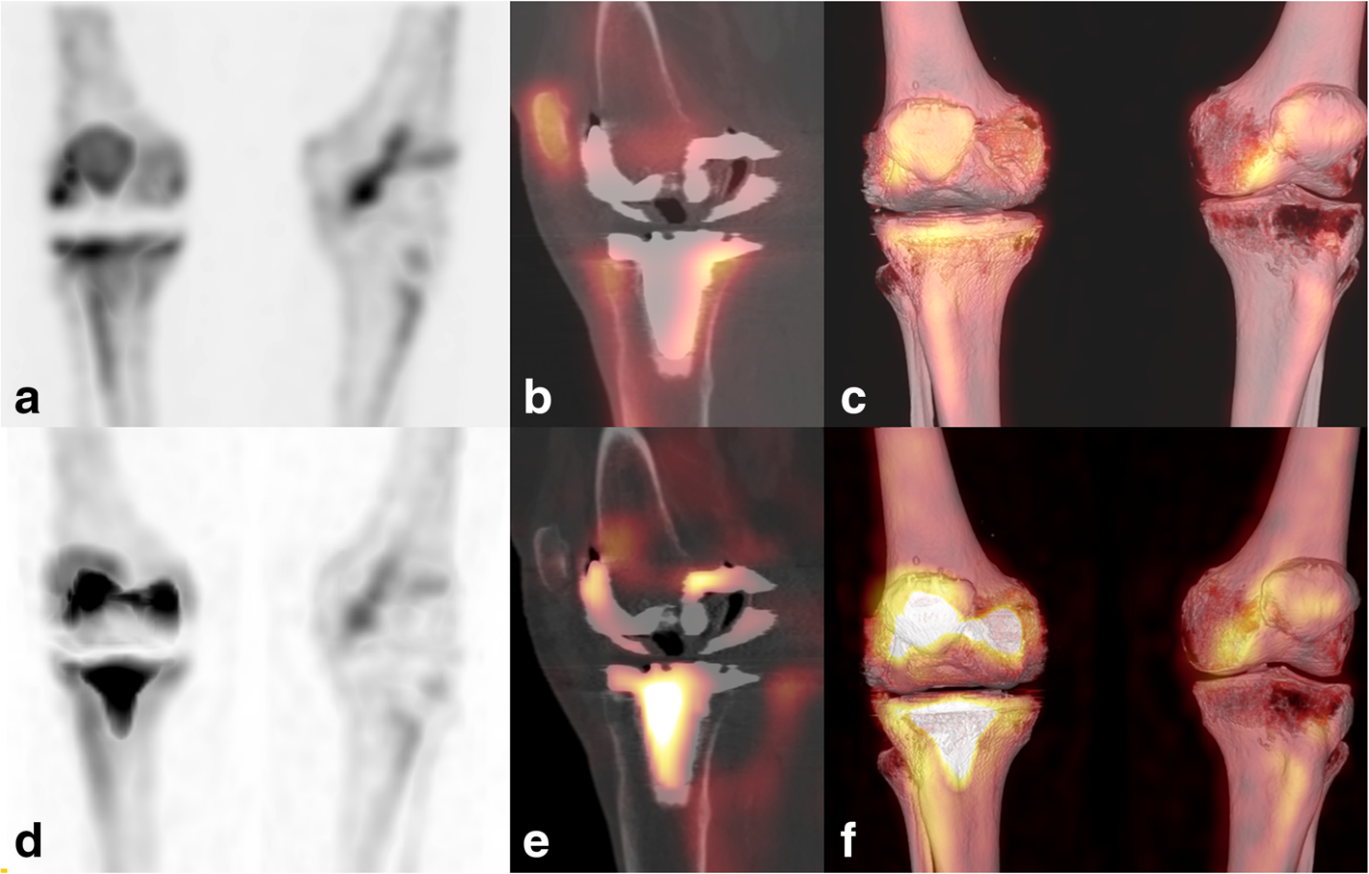
xSPECT Bone “shining metal artefact” in the knee. SPECT/CT images of a 77-year-old female patient 17 years after insertion of a knee arthroplasty. MIP image with xSPECT Bone reconstruction (**d**), fused sagittal SPECT/CT (**e**) and fused 3D reconstruction (**f**) show high “pseudouptake” of the tibial and femoral arthroplasty components. Corresponding images (**a**–**c**) with conventional Flash3D reconstruction show no such abnormal radiotracer uptake in and around the metal implants but only true increased uptake in the adjacent bone and patella. Conservative treatment with physiotherapy was initiated after the SPECT/CT

This report covers five patients who underwent bone scintigraphy with SPECT/CT in our department. The patients were injected with 700 MBq ± 10% (as per national dose reference levels) of [^99m^Tc]-hydroxydiphosphonate (HDP) and scanned after 3 h on a dual-head Siemens Intevo Bold scanner (Siemens Healthineers, Erlangen, Germany) with a 9.5-mm-thick NaI scintillation crystal and low-energy high-resolution (LEHR) collimators. A 15% width energy window was acquired at 140 keV for the primary emission together with a lower scatter window. A low-dose (20 mAs with dose modulation) 130-kVp computed tomography (CT) scan with iterative metal artefact reduction was acquired for the required part of the body, and a SPECT scan was acquired at 60 projections over 180° (15 s per projection in step-and-shoot mode) and a 256 × 256 matrix with isotropic 2.3976 mm pixels. Both xSPECT Bone (24 iterations, 1 subset and 6.00-mm Gaussian filter) and Flash3D (8 iterations, 4 subsets and 6.00-mm Gaussian filter) reconstructions were performed for all patients. While Flash3D reconstructions included scatter, CT-based attenuation and uniformity corrections, xSPECT Bone also included decay and emission correction. In Figs. 1, 2, 3, 4, and 5, three reconstruction images (MIP, fused sagittal SPECT/CT and 3D fused reconstruction) for conventional Flash3D (top images, no artefact) and xSPECT Bone (bottom images with artefact) are reported for different patients and anatomical locations (foot, elbow, shoulder, cervical spine, ribs and knee).

## Conclusions

xSPECT Bone reconstruction often produces the “shining metal artefact” in bones and joints where metallic implants are present. Readers should be aware of this artefact in order to avoid misinterpretation for loosening or infection. We recommend using conventional Flash3D reconstruction without attenuation correction alongside in these cases which is not susceptible for the “shining metal artefact”.

## Data Availability

The authors declare that the data supporting the findings of this study are available within the article.
